# Effects of Black Soldier Fly (*Hermetia illucens*) Larvae Meal Replacement for Fish Meal on Growth Performance, Muscle Quality, Antioxidant Status, Immune Function, and Gut Microbiota in Juvenile Southern Catfish (*Silurus meridionalis*)

**DOI:** 10.3390/antiox14111309

**Published:** 2025-10-30

**Authors:** Huiying Wang, Gao Gao, Jialong Chen, Dan Jia, Qing Hu, Hanqi Duan, Bin Zhang, Run Bi, Qingquan Hu, Baoliang Bi

**Affiliations:** 1 College of Animal Science and Technology, Yunnan Agricultural University, Kunming 650201, China; 2023210428@stu.ynau.edu.cn (H.W.); gaogao@ynau.edu.cn (G.G.); 2024210436@stu.ynau.edu.cn (J.C.); jiadan@ynau.edu.cn (D.J.); huqinggw@ynau.edu.cn (Q.H.); 2 Key Laboratory of Plateau Fishery Resources Protection and Sustainable Utilization, Universities of Yunnan Province, Kunming 650201, China; 3 Yunnan Provincial Academy of Animal Science and Veterinary Medicine, Kunming 650224, China; duanhanqi@sjtu.edu.cn (H.D.); bzhang89@hotmail.com (B.Z.); 4 Menghai County Animal Husbandry and Veterinary Workstation, Xishuangbanna Dai Autonomous Prefecture 666100, China; blrun2025@ynau.edu.cn; 5 International College, Yunnan Agricultural University, Kunming 650201, China

**Keywords:** black soldier fly larvae meal, southern catfish, growth performance, gut microbiota, antioxidant capacity, muscle quality

## Abstract

This study evaluated the effects of feeding juvenile southern catfish (*Silurus meridionalis*) with one of six isonitrogenous and isoenergetic diets where fish meal (FM) was replaced by black soldier fly larval meal (BSFLM) at 0%, 10%, 20%, 30%, 40%, and 50% levels on growth, muscle quality, antioxidant capacity, immune response, and gut microbiota of juvenile southern catfish (*Silurus meridionalis*). A total of 1620 fish (9.20 ± 0.15 g) were fed one of six experimental diets for 8 weeks. Results demonstrated that a 50% replacement (H50 group) significantly improved weight gain rate, specific growth rate, and protein efficiency ratio (*p* < 0.001). Antioxidant capacity (T-AOC) was enhanced in groups H30 and H50, while immune markers lysozyme (LZM) and alkaline phosphatase (AKP) showed mixed responses. Muscle texture properties such as chewiness and adhesiveness were significantly altered across treatments. Gut villi remained structurally intact in all groups, and liver histology appeared normal. No significant differences were found in muscle amino acid or fatty acid profiles. Gut microbiota analysis revealed shifts in microbial composition, with increased abundance of Clostridia and Escherichia and functional enrichment in metabolic pathways at higher substitution levels. Interspecies network analysis indicated potential cooperation among beneficial microbes through metabolite exchange. It is concluded that 50% BSFLM substitution optimizes growth performance, muscle quality, and antioxidant capacity, while modulating gut microbiota, indicating its promise as a sustainable FM alternative and functional ingredient in aquafeeds.

## 1. Introduction

Aquaculture stands as one of the most dynamic sectors in the global food system ([1]). Over the past half-century, global per capita fish consumption has nearly doubled in edible weight terms, now comparable to poultry and pork [2,3]. The expansion of aquaculture production has both met and stimulated growing demand for fish, offering a healthier and more environmentally sustainable alternative to red meat consumption [4]. Notably, aquaculture output growth has outpaced other food commodities by twofold over the past 25 years, evolving into a mature international industry [1,5]. Inland freshwater aquaculture dominates the sector, contributing 75% of global edible weight production in 2020 [6]. Asia remains the largest producer, accounting for 92% of global aquaculture output in 2020, with China alone contributing 57% of total production volume and 59% of global value [3]. This sector provides a critical food source for humanity.

Recent years have witnessed growing interest in insect protein as an alternative protein source for aquafeeds, driven by global fishmeal shortages, price volatility, and sustainability concerns [7]. Black Soldier Fly (*Hermetia illucens*) Larvae Meal (BSFLM) emerges as a promising fishmeal substitute due to its high protein content (36.2–62.7%), balanced amino acid profile, and efficient resource conversion capabilities [8]. The larvae’s rapid reproductive cycle, abundant protein-lipid composition, and essential amino acid pattern resembling fishmeal position it as a valuable animal protein source [9]. Studies demonstrate that insect meals can partially or fully replace fishmeal in crustacean and fish diets without compromising growth performance, feed utilization, intestinal health, immunity, or muscle quality, with some formulations even enhancing these parameters [10]. Among insect-derived proteins, BSFLM shows particular promise for aquafeed applications. Compared to other insect meals like mealworm (*Tenebrio molitor*), BSFLM often demonstrates a superior nutritional profile, particularly in terms of its lauric acid (C12:0) content, which has known antimicrobial and health-promoting properties, and its ability to be reared efficiently on organic waste streams, enhancing its sustainability credentials.

Beyond nutritional substitution, BSFLM contains bioactive components (e.g., antimicrobial peptides, chitin) that improve fish health. For instance, replacing 30% of fishmeal with BSFLM in Banded Catfish (*Pseudobagrus fulvidraco*) juveniles significantly reduced serum alanine aminotransferase activity while enhancing antioxidant capacity [11]. Similarly, supplementing 1% BSFLM paste in Largemouth Bass (*Micropterus salmoides*) diets improved growth performance, disease resistance, antioxidant activity, and intestinal health, highlighting its efficacy as a functional feed additive [12]. Furthermore, BSFLM’s favorable fatty acid profile and amino acid composition (e.g., high lysine and methionine content) optimize feed nutritional structure, with partial substitution shown to increase whole-body crude protein content in fish [13]. These findings position BSFLM as both a nutritional alternative and a functional feed enhancer.

Previous studies have indicated that high dietary inclusion levels of black soldier fly larvae meal (BSFLM) as a fish meal (FM) substitute can induce adverse effects on the growth and health of aquatic animals. For instance, in juvenile hybrid crucian carp (*Carassis auratus*), weight gain rate and feed utilization efficiency significantly decreased when the replacement level exceeded 40% [14]. Furthermore, in species like sea bass, a high substitution rate (e.g., 50%) has been associated with histopathological alterations, including liver damage and intestinal injury [15]. Based on this collective evidence, and to ensure robust growth performance and health status of the farmed animals, the present study was designed to investigate a relatively safe and practical replacement range of 0% to 50% to determine the optimal inclusion level of BSFLM in diets for juvenile southern catfish (*Silurus meridionalis*).

The Southern Catfish (*Silurus meridionalis*) has gained significant popularity in Chinese markets due to its delicate texture, high nutritional value, and premium market positioning [16]. This species has emerged as one of the fastest-growing specialty fish in pond aquaculture over the past decade, valued for its high fecundity, stress tolerance, and efficient feed conversion [17]. While BSFLM has been investigated in other siluriformes, such as the African catfish (*Clarias gariepinus*) and its hybrids, the effects on the commercially important Southern Catfish remain unexplored. Furthermore, there is a lack of focused studies on the critical juvenile stage (e.g., the 9.20 ± 0.15 g size class used in this trial), where nutritional programming is pivotal for subsequent growth and health. Therefore, this study aims to fill this knowledge gap by systematically evaluating the effects of replacing 0% to 50% of dietary fishmeal with BSFLM on the growth performance, muscle quality, antioxidant capacity, immune response, and gut microbiota of juvenile Southern Catfish. The findings will provide a scientific basis for the application of BSFLM as a sustainable and functional aquafeed ingredient for this species.

## 2. Materials and Methods

### 2.1. Animal Ethics Statement

The animal management procedures followed the guidelines of the Committee on Ethics of Animal Experiments at Yunnan Agricultural University (No. 202504003). The animal experiments comply with the ARRIVE guidelines.

### 2.2. Experimental Fish, Diets and Conditions

Six isonitrogenous (approximately 42.78% crude protein) and isoenergetic (15.63 MJ/kg) experimental diets (labeled H0, H10, H20, H30, H40, and H50) were formulated by replacing 0%, 10%, 20%, 30%, 40%, and 50% of fishmeal (FM) with 0%, 5%, 10%, 15%, 20%, and 25% Black Soldier Fly Larvae Meal (BSFLM), respectively. Detailed diet formulations and nutritional compositions are provided in Table 1.

The experimental black soldier fly larvae meal was provided by Yunnan Provincial Animal Husbandry and Veterinary Research Institute, and was tested to contain 44.72% crude protein, 19.83% crude fat, 9.60% crude fiber, and the amino acid composition is shown in Table 2.

All diets were processed at the Laboratory of Animal Science and Technology, Yunnan Agricultural University. Ingredients were ground using a universal grinder and sieved through a 60-mesh screen. Vitamin–mineral premixes were homogenized via stepwise dilution, followed by the addition of fish oil, soybean oil, lecithin, and water. The inclusion levels of fish oil and soybean oil were adjusted across the dietary treatments to compensate for the varying lipid content introduced by the BSFLM and to maintain all diets at isoenergetic levels. This ensured that differences in growth and physiological responses could be more confidently attributed to the protein source itself rather than to disparities in dietary energy. The mixture was pelleted into 2.0 mm diameter granules using an SLX-80 twin-screw extruder (Baker Perkins, Peterborough, UK). The feed material was air-dried at 25 °C until the moisture content was below 10%, before being reserved at −20 °C in a refrigerator for later use. The amino acid composition is shown in Table 2. The amino acid composition of the experimental diets was determined using an automatic amino acid analyzer (Biochrom 30+, Biochrom, Cambridge, UK) after acid hydrolysis with 6 mol/L HCl (reflux for 23 h at 110 °C).

### 2.3. Facilities, Fish and the Feeding Trial

The experiment was conducted in a recirculating aquaculture system (RAS) with a total water capacity of 36,030 L, comprising water treatment, oxygenation, and real-time water quality monitoring modules. Eighteen square net cages (1.0 m× 1.0 m × 1.5 m; 1500 L each) were installed within the system. Juvenile southern catfish (*Silurus meridionalis*) were obtained from Chengdu Meicheng Aquatic Products Co., Ltd. (Chengdu, China). Prior to the trial, the fish were acclimatized for 14 days in the RAS facility of the Yunnan Institute of Animal Science and Veterinary Research. A total of 1620 healthy individuals (mean body weight: 9.20 ± 0.15 g) were selected and randomly allocated into the cages at a density of 90 fish per cage. The 8-week trial involved twice-daily satiation feeding at 09:30 and 18:30. The amount of diet fed in the first week was 3% of the southern catfish ‘s starting weight. According to the feeding situation and the estimated weight of the southern catfish, the feeding amount was adjusted once per week. Throughout the experiment, feed intake, mortality, and water quality parameters were monitored, including dissolved oxygen (DO: 6.75 ± 0.61 mg/L), temperature (22.85 ± 2.42 °C), pH (8.7 ± 0.2), nitrite (NO_2_^−^: 0.029 ± 0.019 mg/L), and ammonia nitrogen (NH_3_-N: 0.13 ± 0.05 mg/L).

### 2.4. Sampling

After the experiment, fish were fasted for 24 h before sampling. The weight gain rate (WGR) and survival rate (SR) were measured for each group. Twenty fish were randomly selected from each tank, anesthetized with 3-EBMS, and blood was collected from the caudal vein to prepare serum (centrifuged at 3500× *g* for 10 min). The serum was stored at −80 °C for biochemical and enzyme activity analysis. After dissection, intestinal, liver, and dorsal muscle tissues were collected. Five samples were fixed in 4% paraformaldehyde for paraffin section preparation and histological observation. Fifteen intestinal and liver samples were stored at −80 °C for 16S rRNA sequencing and enzyme activity analysis. Fresh dorsal muscle samples were used for texture profile analysis (TPA), pH measurement, and nutritional composition analysis (moisture, crude protein, fatty acids, and amino acid composition). All analyses were conducted following standard experimental protocols. When calculating the growth performance indicators, all the weight data are in terms of wet weight. The growth indices were calculated as follows:

Survival rate (SR, %) = (Final number of fish/Initial number of fish) × 100

Weight gain rate (WGR, %) = (Final body weight − Initial body weight)/Initial body weight × 100

FI (g/fish) = Σ[(Feed offered − Uneaten feed)]/Number of fish

Specific growth rate (SGR, %/d) = (ln Final body weight − ln Initial body weight)/Number of experimental days × 100

Feed efficiency (FE, %) = (Final body weight − Initial body weight)/Feed intake

Feed coefficient (FCR) = feed intake/(final fish weight − initial fish weight)

Protein efficiency rate (PER, %) = (Final body weight − Initial body weight)/(Feed intake × Crude protein content of feed) × 100

HSI (%) = (Liver weight/Body weight) × 100

VSI (%) = (Viscera weight/Body weight) × 100

CF = (Body weight/Body length^3^) × 100

### 2.5. Nutritional Component Analysis Methods

The feed ingredients and experimental fish body composition were analyzed in triplicate according to the corresponding national standards. Moisture content was determined using the 105 °C drying to constant weight method [18] with a hot air forced convection oven (DHG-9070A), Shanghai Yiheng Company, Shanghai, China. Crude protein content was measured using the Kjeldahl nitrogen determination method [19] with a fully automatic Kjeldahl nitrogen analyzer (SKD-1000), Shanghai Peiou Company, Shanghai, China. Crude fat content was determined using the Soxhlet extraction method [20] with petroleum ether as the solvent. Crude ash content was measured using the 550 °C incineration method [21] with a box-type resistance furnace (SX-410), Tianjin Taisite company, Tianjin, China. Feed crude fiber was determined using the filter bag method [22]. Gross energy was determined using an oxygen bomb calorimeter (microcomputer fully automatic calorimeter, ZDHW-5000, Hebi Zhongchuang Instrument Co., Ltd., Henan, China). The composition of muscle amino acids and feed amino acids was analyzed using the Biochrom 30+ Protein Automatic Analyzer (produced by Bio-chrom Company, Cambridge, UK). Sample concentration was calculated by substituting the mass spectrometry peak area of the analyte into the linear equation.

### 2.6. Muscle Texture and Biochemical Analysis

Six muscle samples (1 cm × 1 cm × 0.5 cm; approximately 2 g each) were collected from the dorsal region on the same side of the fish and positioned at the center of the testing platform. A texture profile analysis (TPA) was performed using a Shimadzu texture analyzer (Kyoto, Japan) under the following parameters: a 36 mm cylindrical probe, 10 g trigger force, 1 mm/s pre-test, test, and post-test speeds, and 40% compression ratio. Two consecutive TPA cycles were conducted to measure hardness, springiness, cohesiveness, adhesiveness, chewiness, and resilience. Muscle pH was determined via direct measurement using a PH600 pH meter (Thermo Fisher Scientific, Waltham, MA, USA). For collagen quantification, fresh muscle tissue was homogenized, and hydroxyproline content was analyzed with a commercial assay kit (Nanjing Jiancheng Bioengineering Institute, Nanjing, China). Collagen content was calculated by assuming *hydroxyproline* constitutes 13.4% of total collagen.

### 2.7. Serum Biochemical Indices

Intestinal and hepatopancreatic tissues were homogenized in 0.9% sterile saline solution at a mass-to-volume ratio of 1:9 (tissue weight to saline volume). The homogenate was maintained on ice and centrifuged at 4 °C for 10 min (3000× *g*) using an Eppendorf 5415R centrifuge (Hamburg, Germany). The supernatant was collected for subsequent analyses. The total protein (TP) in the supernatant was quantitatively detected using a commercial assay kit (produced by Jiankang Biotechnology Research Institute in Nanjing, China, Catalog No. A045-2) based on the Coomassie Brilliant Blue method. Serum biochemical parameters, including glucose (GLU, Glucose Oxidase, Catalog No. A154-1-1), albumin (ALB, colorimetric method, Catalog No. A028-1-1), non-esterified fatty acid (NEFA, Microplate method, Catalog No. A042-2-1), globulin (GLO, calculated by subtracting ALB from TP), triglycerides (TG, Spectrophotometry, Catalog No. A110-2-1), total cholesterol (T-CHO, Spectrophotometry, Catalog No. A111-2-1), high-density lipoprotein (HDL-C, Spectrophotometry, Catalog No. A112-2-1) and low-density lipoprotein (LDL-C, Spectrophotometry, Catalog No. A113-2-1) and very low-density lipoprotein (VLDL, calculated as TG/5), were measured using corresponding assay kits (Nanjing Jiancheng Bioengineering Institute, Nanjing, China).

Serum immune markers comprised lysozyme (LZM, turbidimetry, Catalog No. A050-1-1), alkaline phosphatase (AKP, Microplate method, Catalog No. A059-1-1), aspartate aminotransferase (AST, colorimetric method, Catalog No. C010-1-1), and alanine aminotransferase (ALT, Microplate method, Catalog No. C009-2-1). Antioxidant parameters, including superoxide dismutase (SOD, Hydroxylamine method, Catalog No. A001-1), malondialdehyde (MDA, TBA method, Catalog No. A003-1), catalase (CAT, Ammonium molybdate method, Catalog No. A007-1-1), and total antioxidant capacity (T-AOC, FRAP method, Catalog No. A015-3-1), were analyzed following the protocols provided by the Nanjing Jiancheng assay kits (Nanjing Jiancheng Bioengineering Institute, Nanjing, China). The results were calculated according to the formulae within the instructions. The absorbance of the samples was measured using a microplate reader (Thermo-1510, Thermo Fisher Scientific, Waltham, MA, USA).

### 2.8. Histological Section Observation

Segments 2 to 3 cm above the midgut at the same part were removed for histological investigation. The midgut was fixed for 24 h in 4% buffered paraformaldehyde before being dried in a graded ethanol series. Following that, the samples were xylene-infiltrated, paraffin-embedded, and sectioned with a rotary microtome. Hematoxylin-eosin solution (Nanjing Jiancheng Co., Nanjing, China) was used to stain the samples, and photographs were taken using a digital camera fitted to a microscope (Pannoramic MIDI FL., Budapest, Hungary). The soft of Case Viewer 2.0 analysis was used. Liver and muscle tissues were processed similarly. Acquired images were analyzed using ImageJ Launcher software (version 1.54g, National Institutes of Health, Bethesda, MD, USA) to quantify histological parameters.

### 2.9. Gut Microbiota 16S rRNA Analysis

During sampling, intestinal tissue samples were collected from three fish per group (*n* = 3). There were a total of six groups (H0, H10, H20, H30, H40, H50), and samples from 18 fish in total were obtained for microbial community analysis. Prior to DNA extraction, intestinal tissue samples were rinsed with sterile phosphate-buffered saline (PBS) to remove luminal contents and potential contaminants. Total microbial DNA was extracted using the E.Z.N.A.^®^ Soil DNA Kit (Omega Bio-tek, Norcross, GA, USA). DNA quality was assessed by 1% agarose gel electrophoresis, while concentration and purity were determined using a NanoDrop 2000 spectrophotometer (Thermo Fisher Scientific, Waltham, MA, USA). The V3-V4 hypervariable region of the 16S rRNA gene was amplified with primers 338F (5′-ACTCCTACGGGAGGCAGCAG-3′) and 806R (5′-GGACTACHVGGGTWTCTAAT-3′). Amplified products were pooled, purified using the AxyPrep DNA Gel Extraction Kit (Axygen Biosciences, Union City, CA, USA), and quantified with a Quantus™ Fluorometer (Promega, Madison, WI, USA). Sequencing libraries were prepared with the NEXT-FLEX Rapid DNA-Seq Kit (Bioo Scientific, Austin, TX, USA) and sequenced on an Illumina MiSeq PE300 platform (Illumina, San Diego, CA, USA).

Raw sequences were quality-filtered using Fastp software (version 0.23.2) and assembled with FLASH. Operational taxonomic units (OTUs) were clustered at 97% similarity using Uparse, followed by taxonomic classification. Alpha diversity indices (Shannon, Simpson, Chao1, Ace, and Coverage) and beta diversity analyses (principal coordinate analysis [PCoA] and principal component analysis [PCA]) were calculated using Mothur software (version 1.48.0). Functional predictions of microbial metabolism and ecological roles were performed via PICRUSt and FAPROTAX, respectively. Microbial co-occurrence networks were constructed using SparCC and visualized with Gephi software (version 0.10.1).

### 2.10. Data Processing and Analysis

All data were tested for assumptions of normality using the Shapiro–Wilk test [23] and homogeneity of variances using Levene’s test. Data that met these assumptions were subjected to a one-way analysis of variance (ANOVA), followed by Tukey’s honest significant difference (HSD) post hoc test for multiple comparisons among the six dietary treatment groups (H0, H10, H20, H30, H40, H50).

The linear and quadratic polynomial contrasts were applied to assess the trends in the measured parameters across the increasing levels of fish meal replacement by black soldier fly larval meal (BSFLM). A broken-line regression analysis was performed on the weight gain rate (WGR) and specific growth rate (SGR) data to determine the optimal BSFLM inclusion level.

All the above statistical analyses were conducted using SPSS Statistics version 26.0 (IBM, Armonk, NY, USA). Additionally, gut microbiota data were analyzed using R software (version 4.0.3) with appropriate packages for multivariate statistics. Differences were considered statistically significant at *p* < 0.05. The results are presented as the mean ± standard error of the mean (SEM).

## 3. Results

### 3.1. Amino Acid Composition of Black Soldier Fly Larvae Powder, Fish Meal and Diets

Table 2 presents the amino acid contents in black soldier fly larvae powder, fish meal, and the diet. When compared to fish meal, black soldier fly larvae powder contains higher levels of essential amino acids (EAAs), including histidine, lysine, methionine, and phenylalanine, as well as non-essential amino acids (NEAAs) such as tyrosine. With the increasing substitution level of black soldier fly larvae powder, the contents of certain EAAs (histidine, isoleucine, leucine, lysine, methionine, and phenylalanine) and NEAAs (alanine, aspartic acid, glutamic acid, proline, cysteine, and tyrosine) show an upward trend. Conversely, the contents of some NEAAs (glutamic acid, glycine, serine and arginine) decline. Overall, the replacement of fish meal with black soldier fly larvae powder appears to lead to an increase in the total EAA content and a decrease in the total NEAA content.

### 3.2. Growth Performance and Biological Indexes

Following a 56-day feeding trial, all experimental groups exhibited high survival rates (SR). The weight gain rate (WGR), specific growth rate (SGR), and protein efficiency ratio (PER) in the H50 group were significantly higher than those in the control group (H0) (*p* < 0.001). Conversely, the H30 group demonstrated significantly lower WGR and SGR compared to H0 (*p* < 0.001). The feed conversion ratio (FCR) was significantly elevated in the H20 and H30 groups relative to H0, while their PER values were markedly reduced (*p* < 0.001). Feeding rates (FR) in the H20, H30, and H40 groups were significantly higher than those in H0 (*p* < 0.001). The SR revealed a significantly linear relationship (*p* = 0.022), and the WGR (*p* = 0.006), SGR (*p* = 0.007), FCR (*p* = 0.005), FR (*p* = 0.001), PER (*p* = 0.001) revealed a significantly quadratic relationship.

**Figure 1:**
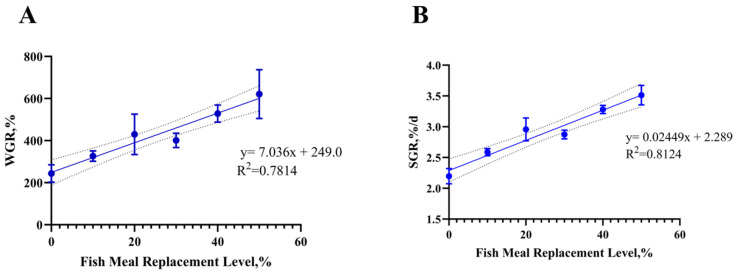
(**A**) Analyzing the linear relationship between the weight gain rate and the fish meal replacement level. (**B**) Analyzing the linear relationship between the specific growth rate and the fish meal replacement level.

All treatment groups showed significantly lower hepatosomatic index (HSI) compared to H0 (*p* < 0.001). Additionally, the viscerosomatic index (VSI) in the H30 and H40 groups was significantly reduced relative to the control (*p* < 0.05). The VSI (*p* = 0.030) and HIS (*p* = 0.010) revealed a significantly linear relationship, and the VSI (*p* = 0.047) and HIS (*p* = 0.006) revealed a significantly quadratic relationship. No significant differences in condition factor (CF) were observed among groups (*p* > 0.05) (Table 3) (Figure 1).

### 3.3. Serum Biochemical Parameters

Serum total protein (TP) and globulin (GLO) levels were significantly reduced in the H20 and H30 groups compared to the control (H0) (*p* < 0.001; Table 4). The content of albumin (ALB) in the H30 group is significantly lower than that in the control group H0 (*p* < 0.05). Low-density lipoprotein (LDL) concentrations in the H10 and H20 groups were significantly higher than those in H0 (*p* < 0.001), whereas LDL levels in the H30 and H40 groups were markedly lower than the control (*p* < 0.001). High-density lipoprotein (HDL) levels in the H30 group were significantly decreased relative to H0, while the H50 group exhibited significantly elevated HDL concentrations (*p* < 0.001). No significant differences in very low-density lipoprotein (VLDL) levels were observed among groups (*p* > 0.05). Non-esterified fatty acids (NEFA) were significantly reduced in the H30, H40, and H50 groups compared to H0 (*p* < 0.001) (Table 4). The NEFA revealed a significantly linear relationship (*p* < 0.001), and the LDL (*p* = 0.003), HDL (*p* = 0.004), TG (*p* = 0.031), VLDL (*p* = 0.031) revealed a significantly quadratic relationship.

### 3.4. Serum Immune Indices

**Figure 2:**
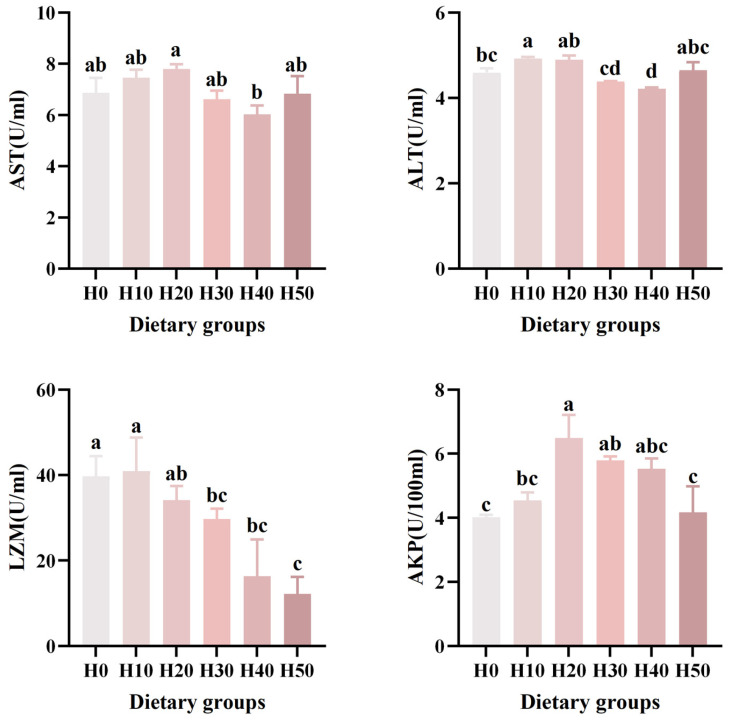
The effects of six different black soldier fly larvae powder diets on the serum levels of AST, ALT, LZM, and AKP in southern catfish. Different letters (a, b, c, d) indicate significant differences among groups (*p* < 0.05).

The immunological analysis revealed significant variations in enzyme activities among dietary groups (Table 5) (Figure 2). Lysozyme (LZM) activity in the H30, H40, and H50 groups was significantly lower than that in the H0 group (*p* < 0.05). Alkaline phosphatase (AKP) activity was significantly elevated in the H20 and H30 groups compared to H0 (*p* < 0.05). No significant intergroup differences were observed for aspartate aminotransferase (AST) activity (*p* > 0.05). Notably, alanine aminotransferase (ALT) activity in the H10 group was significantly higher than in H0, while the H40 group showed a significant decrease relative to H0 (*p* < 0.05). The LZM revealed a significantly linear relationship (*p* < 0.001), and the ALT (*p* = 0.008) revealed a significantly quadratic relationship.

### 3.5. Serum Antioxidant Enzyme Activities

**Figure 3:**
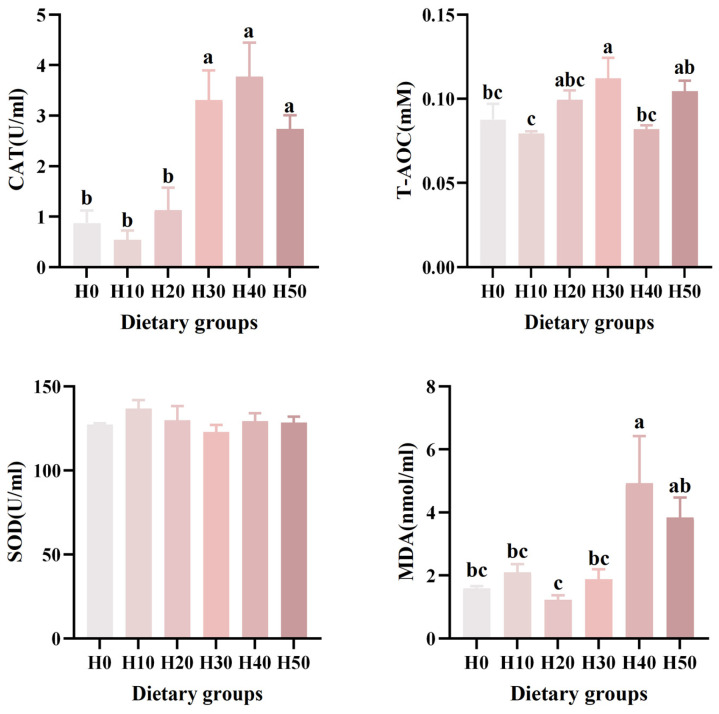
The effects of six different black soldier fly larvae powder diets on the serum levels of CAT, T-AOC, SOD, and MDA in southern catfish. Different letters (a, b, c) indicate significant differences among groups (*p* < 0.05).

No significant differences in superoxide dismutase (SOD) activity were observed among groups (*p* > 0.05). Malondialdehyde (MDA) levels in the H40 group were markedly elevated relative to H0 (*p* < 0.05). Total antioxidant capacity (T-AOC) was significantly enhanced in the H30 group compared to H0 (*p* < 0.05). Catalase (CAT) activity in the H30, H40, and H50 groups was significantly higher than that in the control group (*p* < 0.001) (Table 6) (Figure 3). The MDA (*p* = 0.008) and CAT (*p* < 0.001) revealed a significantly linear relationship, and the T-AOC (*p* = 0.046) revealed a significantly quadratic relationship.

### 3.6. Muscle Quality

There were no significant differences (*p* > 0.05) in moisture, crude fat, crude protein, ash and collagen content between the groups (*p* > 0.05) (Table 7).

The results of texture analysis showed that compared with the control group, the hardness and gumminess of the H20 and H40 groups significantly increased (*p* < 0.001), while the springiness of the H20 and H30 groups significantly decreased (*p* < 0.05). Further observation revealed that the chewiness of the H20, H40 and H50 groups significantly increased, while that of the H10 and H30 groups significantly decreased (*p* < 0.001). In addition, the gumminess of the H20, H40 and H50 groups significantly increased, the adhesion of the H10 group significantly increased, and the recovery ability of the H10, H20 and H40 groups was significantly lower than that of the control group (*p* < 0.05) (Table 8).

**Table 8:** Textural properties of muscle tissue in Juvenile Southern Catfish (*Silurus meridionalis*) (*n* = 3).

Item	Groups						SEM	*p*-Value		
	H0	H10	H20	H30	H40	H50		ANOVA	Linear	Quadratic
Hardness, N	827.56 ^cd^	692.13 ^d^	1403.63 ^a^	662.20 ^d^	1082.52 ^b^	895.78 ^c^	65.002	<0.001	0.579	0.049
Chewiness, mJ	215.88 ^d^	178.18 ^e^	263.99 ^c^	161.13 ^e^	366.49 ^a^	304.96 ^b^	17.893	<0.001	0.008	0.003
Cohesiveness	0.46 ^ab^	0.42 ^b^	0.39 ^b^	0.47 ^ab^	0.53 ^a^	0.51 ^a^	0.015	0.014	0.018	0.580
Gumminess, N	296.82 ^c^	260.64 ^c^	546.74 ^a^	281.28 ^c^	572.56 ^a^	458.61 ^b^	32.180	<0.001	0.020	0.005
Springiness, mm	0.66 ^a^	0.61 ^a^	0.48 ^b^	0.52 ^b^	0.64 ^a^	0.67 ^a^	0.019	<0.001	0.753	0.426
Adhesiveness, mJ	0.01 ^b^	0.01 ^a^	0.02 ^b^	0.01 ^b^	0.01 ^b^	0.01 ^b^	0.001	<0.001	0.370	0.831
Resilience	0.21 ^a^	0.16 ^cd^	0.12 ^d^	0.18 ^abc^	0.17 ^bc^	0.20 ^ab^	0.008	0.001	0.895	0.066

**Figure 4:**
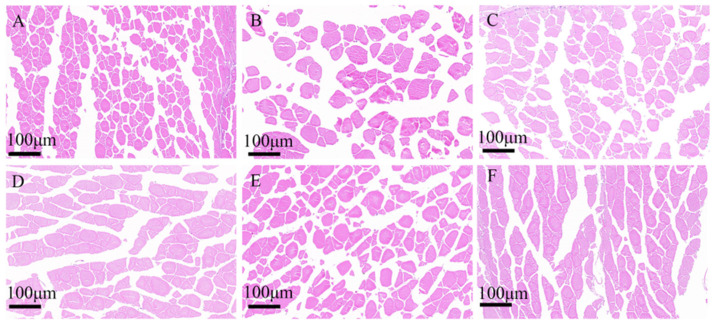
Muscle fiber characteristics of juvenile southern catfish (*Silurus meridionalis*) fed experimental diets for 8 weeks. (**A**) H0 (replacing 0% fishmeal), (**B**) H10 (replacing 10% fishmeal), (**C**) H20 (replacing 20% fishmeal), (**D**) H30 (replacing 30% fishmeal), (**E**) H40 (replacing 40% fishmeal), and (**F**) H50 (replacing 50% fishmeal). Images were captured at 100× magnification, with the scale bar representing 100 µm.

H0: Replaces 0% fishmeal, H10: replaces 10% fishmeal, H20: replaces 20% fishmeal, H30: replaces 30% fishmeal, H40: replaces 40% fishmeal, H50: replaces 50% fishmeal. Data indicate with different letters were significantly different (*p* < 0.05) among treaments.There were no significant differences in the pH values of the muscles in each group. Histology showed that the diameters and areas of muscle fibers in the H10 and H40 groups significantly increased (*p* < 0.001) (Table 9) (Figure 4). The Muscle fiber diameter (*p* = 0.005) and Muscle fiber area (*p* = 0.025) revealed a significantly quadratic relationship.

**Table 9:** Histological characteristics of muscle tissue in Juvenile Southern Catfish (*Silurus meridionalis*) (*n* = 20).

Item	Groups						SEM	*p*-Value		
	H0	H10	H20	H30	H40	H50		ANOVA	Linear	Quadratic
MFD, μm	136.85 ^c^	286.80 ^a^	162.63 ^c^	196.30 ^bc^	250.04 ^ab^	161.21 ^c^	9.968	<0.001	0.826	0.005
MFA, μm^2^	4774.65 ^d^	37,062.45 ^a^	12,279.00 ^cd^	17,674.40 ^bc^	26,078.75 ^ab^	12,824.60 ^cd^	1906.421	<0.001	0.747	0.025

MFD: Muscle fiber diameter; MFA: Muscle fiber area. H0: Replaces 0% fishmeal, H10: replaces 10% fishmeal, H20: replaces 20% fishmeal, H30: replaces 30% fishmeal, H40: replaces 40% fishmeal, H50: replaces 50% fishmeal. Data indicate with different letters were significantly different (*p* < 0.001) among treaments.

### 3.7. Muscle Amino Acid and Fatty Acid Composition

Glutamic acid (Glu), the most potent umami-enhancing natural amino acid, contributes to flavor perception through its sodium salt (monosodium glutamate), a primary component of culinary seasoning. Aspartic acid (Asp), which exhibits secondary umami properties, is commonly utilized as a food flavor enhancer. There were no significant differences in the amino acid content between the groups (*p* > 0.05)(Table 10).

### 3.8. Histological Observation

**Figure 5:**
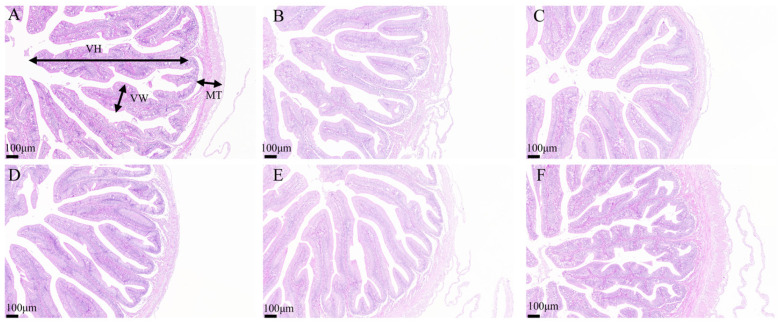
Intestinal tissue characteristics of juvenile southern catfish (*Silurus meridionalis*) fed experimental diets for 8 weeks. (**A**) H0 (replacing 0% fishmeal), (**B**) H10 (replacing 10% fishmeal), (**C**) H20 (replacing 20% fishmeal), (**D**) H30 (replacing 30% fishmeal), (**E**) H40 (replacing 40% fishmeal), and (**F**) H50 (replacing 50% fishmeal). Images were captured at 100× magnification, with the scale bar representing 100 µm. Abbreviations: MT, muscularis thickness; VH, villus height; VW, villus width.

**Figure 6:**
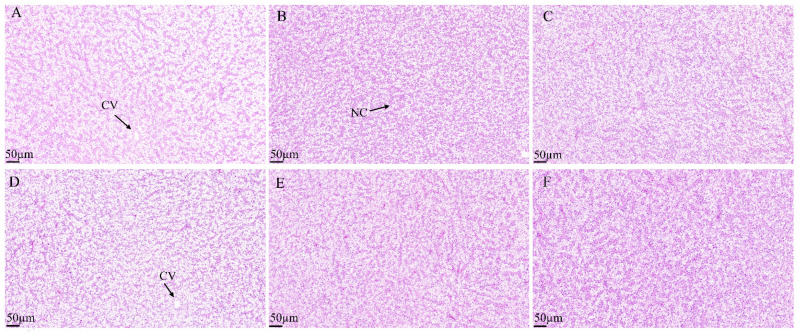
Hepatic tissue characteristics of juvenile southern catfish fed experimental diets for 8 weeks. (**A**) H0 (replacing 0% fishmeal), (**B**) H10 (replacing 10% fishmeal), (**C**) H20 (replacing 20% fishmeal), (**D**) H30 (replacing 30% fishmeal), (**E**) H40 (replacing 40% fishmeal), and (**F**) H50 (replacing 50% fishmeal). Images were captured at 100× magnification, with the scale bar representing 100 µm. Abbreviations: NC, nucleus; CV, vacuole.

Histological analysis of intestinal tissues demonstrated that the intestinal villi in the H0, H30, H40, and H50 groups were structurally intact, tightly arranged, and well-developed (Figure 5). Conversely, the intestinal villi in the H10 and H20 groups exhibited structural disruption and disorganized arrangement. No significant differences in villi length were observed among the groups (*p* > 0.05) (Table 11). Histological examination of liver tissues revealed normal hepatic tissue structure across all experimental groups, with liver cells displaying typical polyhedral morphology. Mild cellular vacuolation and minimal inflammatory cell infiltration were noted; however, no pathological abnormalities were detected (Figure 6).

### 3.9. Analysis of Gut Microbiota

The quality assessment of sequencing data for each sample is shown in Table 12. After sequencing, the average original sequence number (Raw SeqNum) of each sample was the highest in the H10 group (107,811 sequences) and the lowest in the H40 group (91,563.33 sequences). After quality control of the sample data, the proportion of Clean SeqNum to Raw SeqNum for each sample was over 97%. The average effective sequence read length of each group of samples was over 400 bp, which met the sequencing requirements, indicating that this sequencing covered all sequences in the 16S rRNA V3-V4 region. As shown in Table 13, the library coverage (Coverage) of each sample was above 99%, so the sequencing results of this time could represent the real situation of the samples, and all sequences had been detected. The Shannon index was highest in the H40 group, while the Simpson index was lowest, but there were no significant differences between the experimental groups (*p* > 0.05). The Shannon (*p* = 0.030) revealed a significantly linear relationship.

**Figure 7:**
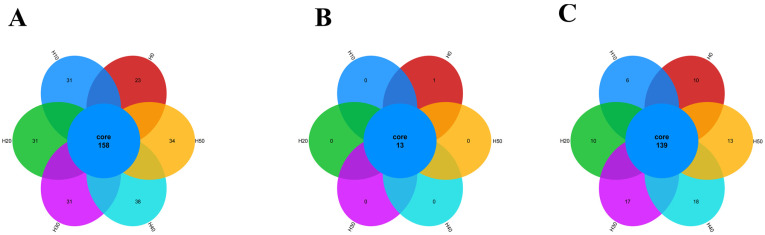
Species distribution radar plots: (**A**) OTU, (**B**) Phylum, (**C**) Genus.

As shown in Figure 7, there were 158 OTUs in the experimental groups, and the number of unique OTUs was the highest in the H40 group and H50 group, with 38 and 34, respectively; with the increase in the level of black soldier fly larvae powder replacing fish meal in the diet, the number of OTUs gradually increased.

**Figure 8:**
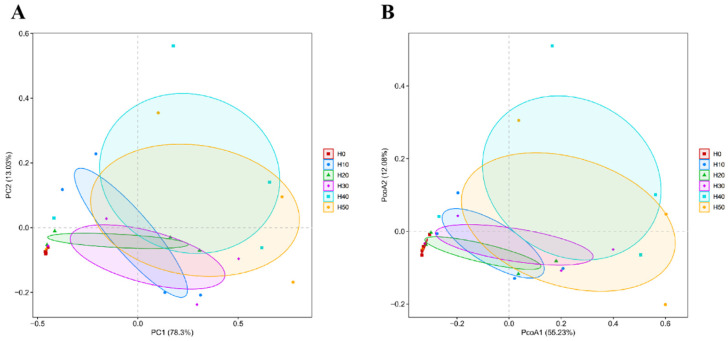
Beta diversity analysis of OTU level: (**A**) PCA, (**B**) PcoA.

**Figure 9:**
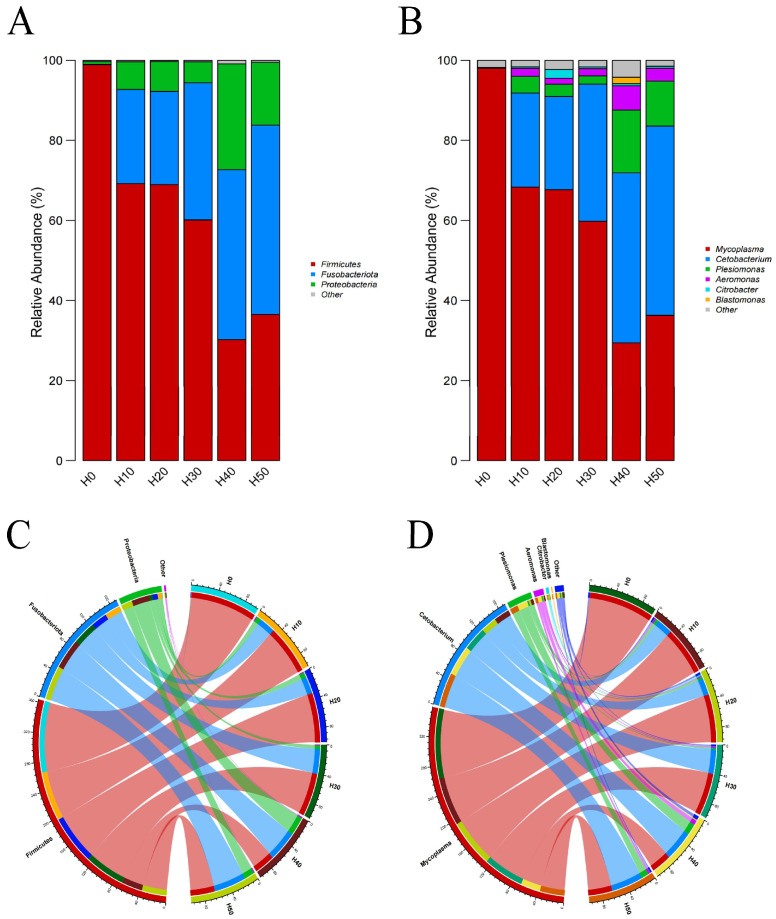
Columnar diagram of relative abundance of species composition at the (**A**) phylum and (**B**) genus levels. Co-linearity diagram of species composition at the (**C**) phylum (**D**) genus levels. In Figure (**C**), red represents *Firmicutes*, blue represents *Fusobacteriota*, green represents *Proteobacteria*, and purple represents Other. In Figure (**D**), red represents *Mycoplasma*, dark blue represents *Cetobacteerium*, green represents *Plesiomonas*, purple represents *Aeromonas*, light blue represents *Citrobacter*, and orange represents *Blastomonas*.

At the Phylum level, there were 13 phyla in total across all the experimental groups, among which the H0 group had one phylum with a unique number of species, while the other experimental groups did not have any unique species. At the Genus level, there were 139 genera across all the experimental groups. The H30, H40, and H50 groups had more unique genera than the control group H0 (Figure 7). As shown in Figure 8, PCA and PCoA analyses at OTU level were employed to assess the β-diversity of gut microbiota. The PCA plot revealed a distinct separation between the H0 group and BSFLM groups, indicating significant differences between control and experimental groups while demonstrating high intra-group homogeneity among experimental replicates. The PCoA plot further showed that the H0 group clustered closely with H20 group but separated distinctly from other experimental groups, with statistically significant differences in community structure (*p* < 0.05).

(Figure 9A) displays the phylum-level analysis of dominant gut microbiota (relative abundance >1%), which primarily consisted of *Firmicutes*, *Clostridium*, and *Proteobacteria*. Notably, *Firmicutes* showed the highest average abundance in the control group (H0), while *Fusobacteria* gradually replaced *Firmicutes* as the predominant phylum with increasing substitution levels of BSFLM. At genus level (Figure 9B), the dominant microbiota included *Mycoplasma*, *Cetobacterium*, *Plesiomonas*, *Aeromonas*, *Citrobacter*, and *Bacillus*. A progressive replacement pattern was observed where *Cetobacterium* gradually superseded *Mycoplasma* as the dominant genus. (Figure 9C,D) quantitatively demonstrate the inverse correlation between black soldier fly larval meal supplementation levels and microbial abundances: *Firmicutes* and *Mycoplasma* exhibited gradual declines in relative abundance, whereas *Clostridium* and *Cetobacterium* showed corresponding increases. These findings further validate the aforementioned structural alterations in gut microbiota composition.

### 3.10. Function Prediction of Gut Microbiota by PICRUSt and Related Analysis

**Figure 10:**
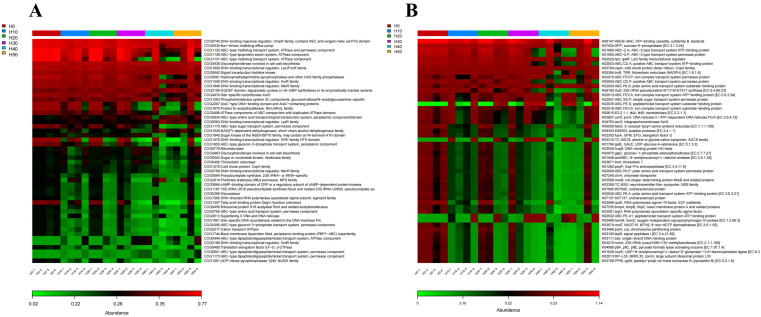
Functional abundance heatmaps based on (**A**) COG (Clusters of Orthologous Groups) and (**B**) KO (KEGG Orthology) database predictions. Heatmaps were generated from functional abundance matrices, where columns represent individual samples, rows represent functional categories, and color intensity reflects normalized abundance values (red: higher abundance; green: lower abundance).

Functional prediction of the gut microbiota was performed based solely on microbial marker gene sequencing data. Using the COG (Clusters of Orthologous Groups) and KO (KEGG Orthology) databases for functional prediction of microbial communities, the analysis revealed that in the black soldier fly additive groups (Figure 10), the functional genes of intestinal microbiota in experimental fish were predominantly activated in multiple aspects, including metabolism, nutrient absorption, and cellular structure/function. Specifically, the H30, H40, and H50 groups showed increased abundance of functional genes related to: Metabolism (COG1028, COG0778, K02495), Material transport (COG1175, COG048, COG0834, COG1653, COG2814, COG0444, K02035, K03310, K02032), Cellular structure and protection (COG0463, COG0492), Signal transduction (COG0834), Substance transportation (COG1744).

**Figure 11:**
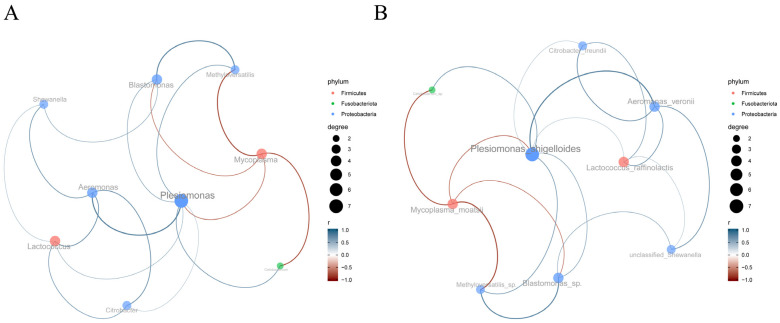
Microbial co-occurrence network analysis based on SPARCC algorithm. (**A**) Genus-level network; (**B**) Species-level network. Key interpretations: Node colors represent distinct bacterial phyla (red: *Firmicutes*; green: *Fusobacteriota*; blue: *Proteobacteria*). Node size corresponds to degree centrality, with larger nodes indicating higher connectivity. Edges (connecting lines) are colored according to the interacting phyla of linked nodes. Edge thickness reflects the absolute value of correlation coefficient (|r|), with thicker edges denoting stronger correlations. Edges were retained only for statistically significant correlations (|r| > 0.6, *p* < 0.05).

The genus-level co-occurrence network (Figure 11A) revealed distinct microbial interaction patterns, with positive correlations observed between *Lactococcus* and *Citrobacter*, *Mycoplasma* and *Cetobacterium*, as well as *Shewanella* and *ethyloversatilis*. Conversely, negative correlations dominated the *Aeromonas-Plesiomonas*, *Blastomonas-Shewanella*, and *Citrobacter-Blastomonas* associations. At the species resolution (Figure 11B), specific symbiotic pairs emerged, including *Lactococcus raffinolactis* with *Citrobacter freundii*, *Mycoplasma moatsii* with *Cetobacterium* sp., and unclassified *Shewanella* with *Methyloversatilis* sp. Antagonistic relationships were particularly evident between *Aeromonas veronii* and *Plesiomonas shigelloides*, *Blastomonas* sp. and unclassified *Shewanella*, as well as *C. freundii* and *Blastomonas* sp.

## 4. Discussion

Currently, the global production of fishmeal and soybean meal is declining, leading to price increases and supply instability. There is an urgent need to find new protein sources that are nutritious, low-cost, and sustainable as alternatives. The black soldier fly larva is considered one of the most promising protein sources to replace fishmeal in aquafeeds and has garnered increasing research attention. Studies have shown that the crude protein content of black soldier fly larvae reaches 47% to 50%, and they are rich in biologically active components such as vitamins, minerals, and amino acids, which can effectively replace fishmeal and soybean meal for use in aquaculture and livestock farming [24].

This study evaluated the growth performance of juvenile southern catfish and found that the H50 group, with 25% BSFLM added, showed significantly higher weight gain rate (WGR), specific growth rate (SGR), and protein efficiency ratio (PER) than the control group (H0). This may be attributed to the rich content of essential amino acids (such as lysine and methionine) in BSFLM, which can fully meet the growth requirements of juvenile southern catfish [25], consistent with the findings of a study where the addition of BSFLM in low-fishmeal diets improved the growth performance of Rainbow Trout (*Oncorhynchus mykiss*) [26]. However, adverse effects on growth performance were observed in the H20 group (with 10% BSFLM added) and the H30 group (with 15% BSFLM added). This indicates that low levels of BSFLM (10% to 15% BSFLM) not only fail to enhance the growth performance and feed conversion rate of juvenile southern catfish but may also have negative impacts, possibly due to the disruption of nutrient balance at these substitution ratios. In previous studies, high substitution levels of 75% to 100% BSFLM were found to improve the growth, feed conversion rate, and survival rate of African catfish (*Clarias gariepinus*), while also reducing feed costs and enhancing profitability [27]. Black soldier fly larval meal can replace up to 60% of fishmeal without affecting the growth, digestive enzyme activity, and whole-fish composition of Striped eel catfish (*Plotosus lineatus*) [11]. Moreover, replacing 25% and 50% of fishmeal had no significant adverse effects on the growth performance and health status of North African Catfish (*Clarias gariepinus*). However, further increasing the fishmeal substitution rate to 100% resulted in significant reductions in growth, feed intake, and protein efficiency ratio [28]. The primary reason for the lack of adverse effects of high-level fishmeal substitution is the high similarity between the amino acid composition of black soldier fly larval meal and fishmeal [29]. However, complete fishmeal substitution can lead to reduced growth rates due to the high concentration of chitin in the feed, which is difficult for fish to digest and results in decreased lipid absorption. Studies have shown that diets containing 4.5% chitin negatively affect the growth performance and nutrient digestibility of rainbow trout [30]. Nevertheless, some studies have indicated that the growth performance of insect meal as a fishmeal substitute varies significantly across different developmental stages (such as embryos, fry, and juveniles) and farming systems in fish [31].

Changes in the hepatosomatic index can reflect liver health, with excessively high values potentially indicating excessive liver fat accumulation [32]. In this experiment, the hepatosomatic index of all experimental groups (H10 to H50) was significantly lower than that of the control group H0. However, histological sections of liver tissue from the experimental groups (H10 to H50) showed slight infiltration in each group, suggesting that black soldier fly meal replacement of fishmeal reduces liver fat accumulation in Sperata aor. This phenomenon may be due to excessive chitin reducing growth and the digestive performance of nutrients such as protein and lipids [33]. Therefore, when considering BSFLM as a substitute for traditional fishmeal, it is essential not only to optimize the substitution ratio but also to study the optimal substitution ratios for different fish species, developmental stages, and farming systems to ensure the best nutritional and economic outcomes.

Texture characteristics are important indicators for evaluating fish meat quality and can be used to compare meat quality across different fish species or farming conditions [34]. This study investigated the effects of BSFLM on the muscle texture and amino acid composition of juvenile southern catfish. The results indicated that adding 25% black soldier fly larval meal (H50) significantly improved the chewiness and adhesiveness of the muscle in juvenile southern catfish, suggesting that a high substitution ratio of 25% black soldier fly larval meal can significantly enhance the taste of fish meat. It has been reported that feeding Half-smooth Tongue-sole (*Areliscus semilaevis*) with 25% defatted black soldier fly larval meal can improve growth performance, feed utilization, and muscle quality [35]. Mikołajczak et al. found that in the juvenile stage of Atlantic salmon (*Salmo salar*), defatted black soldier fly larval meal can replace 29% of fishmeal without negative effects on growth performance and muscle quality [36]. Some studies have suggested that high collagen content typically leads to firmer meat, as collagen provides support and connectivity in muscles. Increased collagen content and cross-linking can make muscle structure more compact and stable, thereby reducing meat tenderness and increasing hardness [37]. Smaller muscle fiber diameters and larger muscle fiber areas usually contribute to improved tenderness, water-holding capacity, and flavor of fish meat, while also enhancing color and taste [38]. However, in this experiment, there were no significant differences in hydroxyproline content, which reflects collagen content, across different treatment groups. Yet, histological sections of muscle tissue observed a significant increase in muscle fiber diameter and area in the H10 (5% BSFLM added) and H40 (20% BSFLM added) groups, which can lead to a tougher texture in fish meat.

The amino acid compositions of muscle are important indicators of nutritional value. Umami amino acids (such as glutamic acid and aspartic acid) are the primary sources of the umami taste in fish meat. The higher the content of these amino acids, the more flavorful the fish meat [39]. This study found that there were no significant differences in the levels of glutamic acid and aspartic acid in the fish meat across the groups, indicating that there were no significant differences in the umami flavor of the fish meat across the groups. However, a meta-analysis of studies using high levels of black soldier fly meal (BSFM) replacement indicated that moderate addition of BSFM can improve fish meat flavor. The study noted that BSFM can serve as an effective alternative to fish meal, with its rich nutritional components helping to enhance meat quality and thereby positively influence fish meat flavor [40].

Changes in serum biochemistry reflect the nutritional metabolism and physiological balance of fish and are considered effective means of assessing the impact of nutritional factors on the health of various fish species [41]. Serum TP, composed of albumin and globulin, reflects the body’s protein metabolism. An increase in TP within a reasonable range indicates enhanced protein synthesis capacity [42]. The results of this study showed that the TP content in the H20 (10% BSFLM added) and H30 (15% BSFLM added) groups was significantly reduced, indicating poor health status in these groups at these substitution ratios. However, studies have shown that adding DBSFLM to the diet of Nile tilapia (*Oreochromis niloticus*) does not affect serum total protein, albumin, globulin, serum alanine aminotransferase, and aspartate aminotransferase levels [43]. High-density lipoprotein helps eliminate cholesterol from the periphery, transporting it to the liver for excretion in bile [44]. In this study, the HDL content in the serum of the H50 group (25% BSFLM added) was significantly increased, indicating that a substitution level of 25% improved the health status of juvenile southern catfish.

Excessive reactive oxygen species free radicals in the body can have adverse effects on health. SOD, CAT, and T-AOC activities are the main indicators for assessing the body’s antioxidant levels [45]. MDA, a product of polyunsaturated fatty acid peroxidation, can damage cells and tissues when accumulated [46]. The results of this study showed that when the BSFLM addition ratio was 15% and 25%, CAT and T-AOC activities were enhanced, indicating improved antioxidant capacity. However, when the BSFLM addition ratio was 20%, serum MDA content was significantly increased, indicating oxidative stress at this substitution ratio. Studies have shown that adding a certain level of black soldier fly can enhance the antioxidant capacity of Pacific White Shrimp (*Litopenaeus vannamei*) [47], and similarly, black soldier fly has a promoting effect on enhancing the liver antioxidant capacity of Largemouth Bass (*Micropterus salmoides*) [48]. This may be related to the antioxidant-active substances (such as melanin and polyphenols) rich in BSFLM [49]. ALT and AST are primarily involved in transamination in the body, and their serum activity increases when liver cells are damaged [50]. Elevated LZM and AKP activities suggest enhanced non-specific immunity, which may stem from the antimicrobial peptide components in BSFLM [51]. The results of this study showed that as the substitution ratio increased, LZM activity did not increase, which differs from the findings of Xiao et al., who observed a 6.8% increase in serum LZM activity in Banded Catfish (*Pseudobagrus fulvidraco*) when black soldier fly replaced 48% of fishmeal [52]. The possible reason is that the substitution ratio was too low to significantly affect LZM activity. This result is consistent with the findings of Zarantoniello et al., who discovered that replacing 25% and 50% of fishmeal in feed with black soldier fly had no significant negative impact on the inflammatory factors and immune response of zebrafish (*Brachydanio rerio*) [53].

Fish growth is closely related to the ability of the digestive system to break down and absorb nutrients, which is influenced by the intestinal absorption area and structural integrity [54]. The results of this study found that after adding black soldier fly larval meal, there were no significant differences in villi length, villus width, and muscle layer thickness across groups, and the intestinal structure of all groups was intact and normal. This result is consistent with the findings of Li et al., who observed that when the level of defatted black soldier fly replacement was between 25% and 50%, the shape of intestinal microvilli was the most regular. At all levels of black soldier fly larval powder substitution, the integrity of the intestinal structure was maintained. These functional benefits were likely mediated by changes in the microbial community rather than by morphological alterations. However, when the replacement level was between 75% and 100%, there was more intestinal tissue debris [55]. This indicates that excessive black soldier fly replacement can damage intestinal tissue, possibly due to the chitin present in DBSFLM feed. Chitin and its derivatives have been reported to reduce the intestine’s ability to absorb nutrients [56].

The composition and metabolic activities of the gut microbiota in aquatic animals are crucial for host health [57]. Alpha diversity analysis reflects the species diversity and abundance of gut microbiota. In this study, as the addition of black soldier fly larval meal increased, the Shannon, Chao1, and Ace indices of the experimental groups also gradually increased. Petal diagrams showed that the number of unique OUTs and the number at the Genus level were higher in the H30, H40, and H50 groups than in the control group H0. This indicates that moderate addition of BSFLM not only enhanced the diversity of gut microbiota but also maintained the overall abundance. Principal component analysis (PCoA) further showed significant differences in gut microbial structure between the BSFLM groups and the control group H0.

The dominant gut microbiota of the experimental fish mainly consisted of three phyla: *Firmicutes*, *Fusobacteriota*, and *Proteobacteria*, which is consistent with the results of a previous study by Pang et al. on the gut microbiota of southern catfish fed different diets [58]. As the addition ratio of black soldier fly larval meal increased, the abundance of *Firmicutes* and *Mycoplasma* gradually decreased, while the abundance of *Fusobacteriota* and *Cetobacterium* gradually increased, indicating that the addition of black soldier fly larval meal may have altered the intestinal environment of the fish. Some bacteria in *Firmicutes* may be more sensitive to environmental changes, and their abundance decreases when the intestinal environment is no longer suitable for their growth [59]. Some bacteria in *Fusobacteriota* are closely related to bile acid metabolism [60]. The addition of black soldier fly larval meal may have affected the synthesis and excretion of bile acids in fish [61], thereby promoting the growth of related bacteria in *Fusobacteriota*. Lauric acid, the primary saturated fatty acid in black soldier flies, can account for up to 50% of total fatty acids and has antiviral, anti-inflammatory, and antibacterial properties [62], which may inhibit *Mycoplasma* and lead to a decrease in its abundance. *Cetobacterium* bacteria have strong environmental adaptability and can utilize a variety of nutrients for growth [63]. When the intestinal environment changes, *Cetobacterium* bacteria may adapt to the new environment more quickly than other bacteria, resulting in an increase in their abundance.

As the proportion of black soldier fly larval meal increased, the variety of COG functional genes gradually increased, with more functional genes related to specific metabolic pathways and regulatory mechanisms appearing. This was mainly reflected in the increase in the abundance and variety of functional genes, as well as the activation of specific functional genes, such as those related to metabolism, material transport, cellular structure and protection, and substance transport. KEGG annotation indicated that these genes were associated with ABC transport systems, amino acid transport and metabolic pathways, and pathways related to amino acid transport and metabolism. Studies have shown that black soldier fly larval meal is rich in antimicrobial peptides, which may activate the expression of antimicrobial peptide-related genes in fish, enhancing resistance to specific pathogens, improving survival rates, and boosting growth performance [64]. However, the activated genes in this study did not include antimicrobial peptide-related genes. These changes may be related to the effects of black soldier fly larval meal on fish metabolism and regulatory mechanisms, providing important clues for further research on the mechanisms of action of black soldier fly larval meal [61].

The interspecies interaction network within the gut microbiota revealed *Plesiomonas* as a hub species with high connectivity, linking multiple microbial taxa. This topological feature suggests its potential role in maintaining network stability and functional integrity. The coexistence of positive and negative correlations indicates complex synergistic and competitive relationships among gut microorganisms. Positive associations may reflect mutualistic relationships, exemplified by the significant correlation between *Plesiomonas* and *Lactococcus* (*p* < 0.01) [65]. As a beneficial genus, *Lactococcus* contributes to intestinal homeostasis through lactate production, which maintains an acidic microenvironment, inhibiting pathogen proliferation [66]. This synergy potentially enhances *Plesiomonas*’ colonization and survival capabilities via metabolic cross-feeding. Notably, the observed coexistence of potentially beneficial (e.g., *Lactococcus*) and opportunistic pathogenic taxa (e.g., *Citrobacter*) in positive associations suggests host-mediated regulation of microbial dynamics through immune or metabolic modulation. Such balanced interactions likely maintain microbial diversity while preventing pathogenic overgrowth, particularly evidenced in healthy subjects where *Plesiomonas* coexists with *Aeromonas* and *Lactococcus* without clinical manifestations. Negative correlations (e.g., *Aeromonas-Plesiomonas* interaction) may drive niche partitioning through resource competition, as demonstrated in piscine models where the *Plesiomonas-Mycoplasma* antagonism maintains microbial equilibrium. Metabolic interdependencies, including *Lactate-Citrate* metabolic cross-feeding and vitamin biosynthesis networks, appear to mediate these interphylum relationships. This delicate balance prevents microbial dominance while ensuring ecosystem resilience, with dysbiosis potentially triggering adverse host outcomes [67].

## 5. Conclusions

This study concludes that replacing fish meal (FM) with black soldier fly larval meal (BSFLM) at a level of 50% is optimal for juvenile southern catfish, as it significantly improves growth performance (weight gain rate and specific growth rate), enhances antioxidant capacity, and beneficially modulates the gut microbiota. Although some beneficial effects on immune markers (lysozyme) and myofibrillar protein content were observed at lower replacement levels (25–30%), the superior growth advantage at the 50% level makes it the most effective replacement strategy. Therefore, a 50% replacement of FM with BSFLM is recommended as a sustainable and functional aquafeed ingredient to enhance productivity in southern catfish farming.

## Figures and Tables

**Table 1:** Formulation and proximate composition of diets (%, dry matter).

Item	Group					
	H0	H10	H20	H30	H40	H50
Ingredient (%)						
Fishmeal ^1^	30.00	27.00	24.00	21.00	18.00	15.00
BSFLM ^2^	0.00	5.00	10.00	15.00	20.00	25.00
Soybean meal	30.00	30.00	30.00	30.00	30.00	30.00
Peanut meal	12.00	12.00	12.00	12.00	12.00	12.00
Wheat bran	7.00	7.00	7.00	7.00	7.00	7.00
Wheat flour	12.00	10.70	9.40	8.10	6.80	5.50
Fish oil	0.00	0.30	0.60	0.90	1.20	1.50
Soybean oil	5.00	4.00	3.00	2.00	1.00	0.00
Soybean lecithin	1.00	1.00	1.00	1.00	1.00	1.00
Ca (H_2_PO_4_)_2_	1.20	1.20	1.20	1.20	1.20	1.20
NaCl	0.28	0.28	0.28	0.28	0.28	0.28
Vitamin C	0.02	0.02	0.02	0.02	0.02	0.02
Choline chloride	0.50	0.50	0.50	0.50	0.50	0.50
Vitamin premix ^3^	0.50	0.50	0.50	0.50	0.50	0.50
Mineral premix ^4^	0.50	0.50	0.50	0.50	0.50	0.50
Total	100.00	100.00	100.00	100.00	100.00	100.00
Chemical composition						
DM, %	90.80	91.00	91.30	91.57	91.97	91.80
OM, %	91.34	91.15	91.20	90.81	90.51	90.43
CP (N × 6.25), %	42.82	43.28	42.36	42.27	42.89	42.90
EE, %	14.31	14.43	14.57	14.63	14.96	15.41
Energy, MJ/kg	15.42	15.93	15.84	15.51	15.46	15.65
CF, %	2.69	3.58	4.02	4.69	4.98	5.80

DM = dry matter; OM = Organic Matter; CP = crude protein; EE = ether extract; CF = crude fiber. ^1^ Fishmeal: Peruvian super steam fishmeal, supplied by Ningbo China Tianbao Grain & Feed Trading Co., Ltd., Ningbo, China. A total of 69.98% crude protein, 9.12% crude fat. ^2^ BSFLM: Dried *Hermetia illuce* larvae provided by Yunnan Academy of Animal Science and Veterinary Sciences. ^3^ Vitamin premix (Foshan, China) (per kilogram of diet): vitamin A 2,000,000 IU, vitamin D_3_ 1,000,000 IU, vitamin E 15,000 mg, vitamin K_3_ 4500 mg, vitamin B_1_ 900 mg, vitamin B_2_ 1440 mg, vitamin B_6_ 9000 mg, vitamin B_12_ 10 mg, vitamin C 10,000 mg, folic acid 5 mg, niacin 100 mg, inositol 300 mg, nicotinic acid 1000 mg, folic acid 60 mg, biotin 1.2 mg. ^4^ Mineral premix (Foshan, China) (per kilogram of diet): FeSO_4_ 15,000 mg, MgSO_4_ 1500 mg, ZnSO_4_ 10,000 mg, CuSO_4_ 6500 mg, MnSO_4_ 6000 mg, KCl 500 mg, NaCl 1000 mg, CaCO_3_ 5000 mg.

**Table 2:** Amino acid composition of FM, BSFLM and diets (mg/g, dry matter).

Amino Acid	Ingredients		Diets					
	FM	BSFLM	H0	H10	H20	H30	H40	H50
EAA								
Histidine	6.61	14.87	7.877	8.43	8.85	9.07	9.67	10.05
Isoleucine	26.26	16.11	15.73	14.50	13.82	13.00	12.75	11.63
Leucine	44.52	28.22	28.20	27.55	26.69	26.32	25.74	25.21
Lysine	20.67	26.63	16.66	17.89	18.35	18.95	19.04	19.63
Methionine	7.17	7.54	4.55	4.87	5.10	4.98	5.49	5.26
Phenylalanine	28.56	39.30	19.31	20.37	22.38	22.62	23.00	24.11
Threonine	27.91	17.45	15.86	14.56	13.32	12.08	11.86	10.60
Valine	34.22	21.94	19.62	18.21	17.72	17.49	17.05	16.66
∑EAA	195.92	172.06	127.81	126.38	126.23	124.51	124.60	123.15
NEAA								
Alanine	33.21	25.86	18.60	17.58	16.53	16.04	15.68	15.01
Aspartic acid	44.71	41.72	37.41	37.90	37.46	36.52	36.31	35.48
Glutamic acid	84.11	56.21	72.24	71.01	70.18	69.17	68.51	67.78
Glycine	51.93	22.45	27.55	26.35	26.43	25.21	24.97	24.39
Serine	57.70	18.44	28.62	27.67	26.82	25.30	24.54	23.73
Arginine	40.85	23.40	30.57	29.56	28.47	27.13	26.87	26.83
Cysteine	6.45	2.67	5.02	5.17	4.89	4.77	4.56	4.49
Tyrosine	20.15	30.81	14.77	15.84	17.14	17.81	18.09	18.95
∑NEAA	339.10	221.56	234.76	231.08	227.92	221.95	219.53	216.66

FM: fish meal; BSFLM: black soldier fly larvae meal; EAA: essential amino acids; NEAA: non-essential amino acids.

**Table 3:** Growth performance, feed utilization efficiency and morphological parameters of Juvenile Southern Catfish (*Silurus meridionalis*) fed experimental diets for 8 weeks (*n* = 3).

Item	Groups						SEM	*p*-Value		
	H0	H10	H20	H30	H40	H50		ANOVA	Linear	Quadratic
SR, %	88.15	94.81	97.78	95.56	98.15	97.78	1.239	0.146	0.022	0.299
FI, g/fish	52.07 ^a^	448.01 ^ab^	446.56 ^b^	447.63 ^ab^	446.36 ^b^	46.56 ^b^	0.71	0.156	<0.001	0.027
WGR, %	243.9 ^d^	326.19 ^cd^	429.45 ^bc^	400.77 ^c^	528.32 ^ab^	620.978 ^a^	32.970	<0.001	<0.001	0.006
SGR, %/d	2.20 ^d^	2.59 ^c^	2.96 ^bc^	2.87 ^c^	3.28 ^ab^	3.51 ^a^	0.113	<0.001	0.770	0.007
FCR	2.39 ^a^	1.79 ^b^	1.51 ^bc^	1.54 ^bc^	1.17 ^cd^	0.98 ^d^	0.120	<0.001	0.161	0.005
PER, %	125.30 ^d^	165.67 ^cd^	200.51 ^bc^	190.99 ^c^	251.77 ^b^	319.30 ^a^	16.376	<0.001	0.950	0.001
VSI, %	12.41 ^a^	8.95 ^bc^	10.12 ^ab^	7.56 ^cd^	6.51 ^d^	11.19 ^ab^	0.556	0.025	0.030	0.047
CF, g/cm^3^	0.79	0.81	0.83	1.1	0.99	0.99	0.028	0.234	0.149	0.514
HSI, %	6.81 ^a^	5.51 ^b^	5.67 ^b^	3.47 ^c^	3.70 ^c^	5.31 ^b^	0.300	<0.001	0.010	0.006

SR: Survival rate; FI: Feed intake; WGR: Weight gain rate; SGR: Specific growth rate; FCR: Feed conversion ratio; PER: Protein efficiency ratio; CF: Condition factor; HSI: Hepatosomatic index; VSI: Viscerosomatic index. Dietary treatments: H0 (0% fishmeal substitution), H10 (10% substitution), H20 (20% substitution), H30 (30% substitution), H40 (40% substitution), H50 (50% substitution). Data indicate with different letters were significantly different (*p* < 0.05) among treaments.

**Table 4:** Serum biochemical indices of Juvenile Southern Catfish (*Silurus meridionalis*) (*n* = 3).

Item	Groups						SEM	*p*-Value		
	H0	H10	H20	H30	H40	H50		ANOVA	Linear	Quadratic
GLU, mmol/L	2.92	3.19	4.00	2.48	3.47	3.42	0.219	0.498	0.698	0.947
TP, g/L	28.39 ^a^	28.70 ^a^	21.63 ^b^	12.49 ^c^	28.88 ^a^	28.88 ^a^	1.510	<0.001	0.849	1.000
LDL, mmol/L	0.43 ^c^	0.52 ^ab^	0.59 ^a^	0.16 ^e^	0.34 ^d^	0.48 ^bc^	0.035	<0.001	0.371	0.003
HDL, mmol/L	1.62 ^bc^	1.75 ^b^	1.51 ^c^	0.74 ^d^	1.72 ^bc^	2.07 ^a^	0.102	<0.001	0.511	0.004
ALB, g/L	7.97 ^ab^	8.24 ^a^	6.13 ^bc^	5.15 ^c^	8.62 ^a^	7.81 ^ab^	0.361	0.006	0.933	0.335
GLO, g/L	20.42 ^a^	20.45 ^a^	15.50 ^b^	7.34 ^c^	20.26 ^a^	21.07 ^a^	1.242	<0.001	0.836	0.584
TG, mmol/L	3.66 ^ab^	4.30 ^ab^	4.66 ^ab^	3.46 ^b^	3.47 ^b^	4.80 ^a^	0.188	0.090	0.626	0.031
NEFA, mmol/L	0.54 ^a^	0.55 ^a^	0.46 ^a^	0.36 ^b^	0.33 ^b^	0.33 ^b^	0.025	<0.001	<0.001	0.947
T-CHO, mmol/L	2.50	2.55	2.78	2.59	2.42	2.49	0.097	0.952	0.747	0.868
VLDL, mmol/L	0.73 ^ab^	0.86 ^ab^	0.93 ^ab^	0.69 ^b^	0.69 ^b^	0.96 ^a^	0.038	0.090	0.626	0.031

Abbreviations: GLU, Glucose (mmol/L); TP, Total protein (g/L); LDL, Low-density lipoprotein (mmol/L); HDL, High-density lipoprotein (mmol/L); ALB, Albumin (g/L); GLO, Globulin (g/L); TG, Triglycerides (mmol/L); NEFA, Non-esterified fatty acids (mmol/L); T-CHO, Total cholesterol (mmol/L); VLDL, Very low-density lipoprotein (mmol/L). Dietary treatments: H0 (0% fishmeal substitution), H10 (10% substitution), H20 (20% substitution), H30 (30% substitution), H40 (40% substitution), H50 (50% substitution). Values are presented as mean ± SD. Data indicate with different letters were significantly different (*p* < 0.05) among treaments.

**Table 5:** Serum immune enzyme activities of Juvenile Southern Catfish (*Silurus meridionalis*) (*n* = 3).

Item	Groups						SEM	*p*-Value		
	H0	H10	H20	H30	H40	H50		ANOVA	Linear	Quadratic
LZM, U/mL	39.70 ^a^	41.00 ^a^	34.18 ^ab^	29.72 ^bc^	16.35 ^bc^	12.21 ^c^	3.296	0.014	<0.001	0.613
AKP, U/100 mL	4.02 ^c^	4.55 ^bc^	6.50 ^a^	5.80 ^ab^	5.54 ^abc^	4.18 ^c^	0.273	0.016	0.598	0.064
AST, U/L	6.87	7.45	7.79	6.62	6.03	6.83	0.205	0.155	0.188	0.224
ALT, U/L	4.59 ^bc^	4.92 ^a^	4.89 ^ab^	4.39 ^cd^	4.22 ^d^	4.65 ^abc^	0.070	0.002	0.107	0.008

LZM = Lysozyme; AKP = Alkaline phosphatase; AST = Aspartate aminotransferase; ALT = Alanine aminotransferase. Diet groups: H0 (0% fishmeal substitution), H10 (10% substitution), H20 (20%), H30 (30%), H40 (40%), H50 (50%). Data indicate with different letters were significantly different (*p* < 0.05) among treaments.

**Table 6:** Serum antioxidant enzyme activities of Juvenile Southern Catfish (*Silurus meridionalis*) (*n* = 3).

Item	Groups						SEM	*p*-Value		
	H0	H10	H20	H30	H40	H50		ANOVA	Linear	Quadratic
SOD, U/mL	127.26	136.82	129.93	122.87	129.45	128.51	1.950	0.531	0.583	0.894
MDA, nmol/mL	1.59 ^bc^	2.10 ^bc^	1.23 ^c^	1.88 ^bc^	4.93 ^a^	3.84 ^ab^	0.399	0.015	0.008	0.285
T-AOC, mM	0.09 ^bc^	0.08 ^c^	0.10 ^abc^	0.11 ^a^	0.08 ^bc^	0.10 ^ab^	0.004	0.040	0.190	0.046
CAT, U/mL	0.87 ^b^	0.54 ^b^	1.13 ^b^	3.31 ^a^	3.78 ^a^	2.74 ^a^	0.341	<0.001	<0.001	0.118

SOD = Superoxide dismutase; MDA = Malondialdehyde; T-AOC = Total antioxidant capacity; CAT = Catalase. H0 (0% fishmeal substitution), H10 (10%), H20 (20%), H30 (30%), H40 (40%), H50 (50%). Data indicate with different letters were significantly different (*p* < 0.05) among treaments.

**Table 7:** Proximate composition analysis of muscle tissue in Juvenile Southern Catfish (*Silurus meridionalis*) (*n* = 3).

Item	Groups						SEM	*p*-Value		
	H0	H10	H20	H30	H40	H50		ANOVA	Linear	Quadratic
DM, %	72.80	73.54	69.71	80.92	71.09	75.57	2.168	0.475	0.703	0.593
Crude fat, %	25.92	20.87	20.43	16.43	20.76	17.50	1.069	0.129	0.029	0.328
CP (N × 6.25), %	74.32	79.55	78.61	80.77	76.83	80.21	0.996	0.181	0.262	0.347
Crude Ash, %	7.42	8.54	7.38	8.96	7.99	8.51	0.622	0.981	0.687	0.836
Collagen, µg/mg wet weight	818.12	605.93	682.16	526.42	568.80	696.96	54.553	0.747	0.452	0.540

DM = dry matter; CP = crude protein. H0: Replaces 0% fishmeal, H10: replaces 10% fishmeal, H20: replaces 20% fishmeal, H30: replaces 30% fishmeal, H40: replaces 40% fishmeal, H50: replaces 50% fishmeal.

**Table 10:** Amino acid content of muscle tissue in Juvenile Southern Catfish (*Silurus meridionalis*) (mg/g) (*n* = 3).

Item	Groups						SEM	*p*-Value		
	H0	H10	H20	H30	H40	H50		ANOVA	Linear	Quadratic
Lysine	66.90	70.75	70.43	72.54	68.65	70.05	0.769	0.425	0.481	0.605
Phenylalanine	30.88	32.87	33.15	34.31	32.45	33.12	0.401	0.262	0.186	0.617
Methionine	18.44	19.63	19.69	20.03	19.01	19.34	0.222	0.403	0.525	0.675
Threonine	33.94	36.40	36.11	36.78	35.65	36.63	0.416	0.413	0.169	0.502
Isoleucine	29.53	31.41	31.59	32.06	30.66	30.99	0.356	0.439	0.467	0.791
Leucine	55.20	58.28	58.36	60.06	57.25	58.37	0.653	0.450	0.294	0.628
Valine	31.16	32.82	32.94	33.71	32.06	32.51	0.370	0.524	0.508	0.737
Aspartic acid	74.68	79.55	79.56	82.05	78.09	79.55	0.916	0.336	0.243	0.640
Serine	33.17	34.72	34.50	36.03	34.31	35.12	0.395	0.490	0.223	0.568
Glutamic acid	132.89	142.40	142.37	146.95	139.49	141.83	1.674	0.275	0.249	0.675
Glycine	33.00	34.74	34.81	36.06	34.43	34.58	0.464	0.653	0.404	0.927
Alanine	43.37	44.85	45.15	47.16	44.97	45.91	0.539	0.528	0.179	0.627
Cysteine	6.24	6.57	6.66	6.35	6.59	6.51	0.053	0.172	0.351	0.578
Tyrosine	26.57	27.81	28.23	28.53	27.30	27.97	0.289	0.467	0.344	0.517
Histidine	18.12	19.41	19.54	20.67	18.96	19.49	0.263	0.108	0.231	0.504
Arginine	44.65	47.24	47.16	47.77	45.64	46.38	0.539	0.625	0.698	0.708
Proline	30.36	32.10	32.35	32.83	30.63	31.43	0.323	0.154	0.837	0.435

H0: Replaces 0% fishmeal, H10: replaces 10% fishmeal, H20: replaces 20% fishmeal, H30: replaces 30% fishmeal, H40: replaces 40% fishmeal, H50: replaces 50% fishmeal.

**Table 11:** Intestinal histomorphological parameters of Juvenile Southern Catfish (*Silurus meridionalis*) (*n* = 3).

Item	Groups						SEM	*p*-Value		
	H0	H10	H20	H30	H40	H50		ANOVA	Linear	Quadratic
IVL, µm	1035.13	952.35	902.47	1062.07	995.79	1143.33	29.848	0.239	0.182	0.149
MT, µm	116.52 ^b^	109.20 ^b^	113.45 ^b^	95.69 ^b^	194.35 ^a^	133.46 ^b^	8.736	0.003	0.080	0.007

IVL: Intestinal villi length; MT: Muscular thickness. H0: Replaces 0% fishmeal, H10: replaces 10% fishmeal, H20: replaces 20% fishmeal, H30: replaces 30% fishmeal, H40: replaces 40% fishmeal, H50: replaces 50% fishmeal. Data indicate with different letters were significantly different (*p* < 0.05) among treaments.

**Table 12:** Intestinal microbiota sequencing data of Juvenile Southern Catfish (*Silurus meridionalis*) (*n* = 3).

Item	Groups						SEM	*p*-Value		
	H0	H10	H20	H30	H40	H50		ANOVA	Linear	Quadratic
Raw SeqNum, reads	101,347.75	108,083.25	116,706.00	101,107.00	97,418.00	113,148.25	4198.260	0.783	0.898	0.322
Clean SeqNum, reads	98,229.50	107,335.75	115,351.75	100,541.50	95,563.75	112,214.75	4190.514	0.739	0.823	0.291
BaseNum, Mb	46,119,756.75	49,589,413.00	53,391,207.25	46,205,830.50	43,912,386.75	51,389,843.75	1,851,262.290	0.716	0.957	0.281
MeanLen, bp	456.40	458.92	457.86	457.39	451.64	455.05	1.389	0.757	0.318	0.511
Effective, %	97.25	99.30	98.70	99.42	97.91	99.14	0.003	0.302	0.368	0.265

Raw SeqNum: Total number of raw sequencing reads obtained from the sequencer. Clean SeqNum: Number of effective sequencing reads after quality control. BaseNum: Number of bases in the Clean SeqNum. MeanLen: Average length of the Clean SeqNum. Effective (%): Percentage of Clean SeqNum relative to Raw SeqNum.

**Table 13:** Alpha Diversity index analysis of sample (*n* = 3).

Item	Groups						SEM	*p*-Value		
	H0	H10	H20	H30	H40	H50		ANOVA	Linear	Quadratic
Shannon	0.16	0.70	0.48	0.61	1.12	0.86	0.112	0.212	0.030	0.483
Chao1	155.43	204.63	150.28	224.26	162.55	231.56	14.308	0.403	0.276	0.182
Ace	161.06	219.68	159.60	241.20	172.36	241.61	15.385	0.396	0.291	0.210
Simpson	0.95	0.66	0.77	0.71	0.52	0.61	0.058	0.396	0.061	0.686
Coverage, %	99.95%	99.94%	99.96%	99.93%	99.95%	99.94%				

H0: Replaces 0% fishmeal, H10: replaces 10% fishmeal, H20: replaces 20% fishmeal, H30: replaces 30% fishmeal, H40: replaces, H50: replaces 50% fishmeal.

## Data Availability

All experimental data generated or analyzed during this research are fully presented in the manuscript.
